# Hybrid deep neural network with PCA based features optimization for enhancing brain tumor classification

**DOI:** 10.1038/s41598-026-39154-7

**Published:** 2026-02-19

**Authors:** Binay Kumar Pandey, Digvijay Pandey, Tsair-Fwu Lee, Mesfin Esayas Lelisho

**Affiliations:** 1 Department of Information Technology, College of Technology, Govind Ballabh Pant University of Agriculture and Technology Pantnagar, Pantnagar, Uttarakhand India; 2 Department of Technical Education Uttar Pradesh, Kanpur, India; 3 Medical Physics and Informatics Laboratory of Electronic Engineering, National Kaohsiung University of Science and Technology, Kaohsiung, Taiwan; 4 Department of Statistics, College of Natural and Computational Science, Mizan-Tepi University, Tepi, Ethiopia

**Keywords:** Brain tumor diagnosis, Deep learning, MRI imaging, DenseNet121, Transfer learning, Principal component analysis, Cancer, Computational biology and bioinformatics, Mathematics and computing, Oncology

## Abstract

Brain tumors have been an important medical concern. This is primarily due to their growth patterns have been hard to predict and their medical needs have been complicated. This work proposes emphasis on a hybrid PCA DenseNet121 convolutional neural network mechanism. The model is intended to improve classification accuracy among four specific classifications: Glioma, Meningioma, Pituitary, and No Tumor. The model incorporates deep features with traditional texture descriptors to obtain an entire set of features. Specifically, it combines the Gray Level Co-occurrence Matrix (GLCM) and Local Ternary Pattern (LTP) with the Color Coherence Vector (CCV). Experimental integrity was a primary focus of this study. To preclude data leakage, the feature extraction and optimization pipeline was strictly partitioned between the training and testing datasets. The implementation of the CCV is methodologically supported by a specific preprocessing stage. In this stage, MRI intensity distributions are quantized into 27 discrete bins. This process classifies pixels as coherent or incoherent based on their spatial connectivity. This method captures subtle relationships between intensity clusters. These associations significantly improve the structural data provided by GLCM as well as LTP. Principal Component Analysis (PCA) has been employed to deal with the resultant feature space in many dimensions. This method preprocesses the feature vector beforehand to the training phase. The PCA transformation has been almost exclusively adapted to the training data, giving them highly significant. This approach eradicates anticipating bias despite ensuring that the model has applicability across diverse situations. The proposed approach has produced an accuracy of 95.89% with regard to classification. This performance has been confirmed by the measurements for the precision, recall, and a F1 score that have retained the same upon 94%. The findings indicate that incorporating multimodal descriptors alongside deep features provides a comprehensive representation of tumor features. This integration minimizes misclassification. It also ensures stable learning patterns throughout the diagnostic process. Additionally, the training and validation processes for each accuracy and loss constantly coincide. This indicates that the model performed an effective task of reducing down on too much excessive overfitting. The elevated degree of performance has been caused by the successful use of dropout normalization. After that, systematic information shuffling enhances the learning process more reliable with an extensive variety of clinical data sets.

## Introduction

The worldwide advancement in healthcare field is essential for augmenting the standard of life and enhanced outcomes for patients. The fundamental objective of this development involves the attempt of enhanced diagnostic accuracy. The increase in accuracy in diagnosis helps both healthcare providers and patients by lowering the possibility of misinterpretation. Furthermore, such precision shields patients from the physical and emotional toll of unnecessary medical procedures^1^. A vital shift in this field has been the rise of high-speed image processing. These technologies provide the immediate and objective results required by modern medicine. The models for deep learning, specifically Convolutional Neural Networks (CNNs), have become very important. This equipment have revolutionized illness identification by allowing healthcare workers to look at intricate medical imaging with unprecedented accuracy^2^. The biological onset of a brain tumor is characterized by cells that cease to follow regulatory signals. In healthy tissue, cellular homeostasis is maintained through a balanced cycle of birth and apoptosis. However, when biological anomalies occur, this balance shatters, leading to the rapid growth of a mass. These tumors are broadly categorized as benign or malignant. While benign tumors tend to remain localized, malignant tumors are aggressive. They invade surrounding healthy tissue in ways that are notoriously difficult to predict; this invasive behavior is clinically defined as cancer^3^. Cancer is capable of damaging any region of the body. Nonetheless, its presence in the brain can be extremely perilous. Brain tumors constitute over 90% of all malignancies within the Central Nervous System (CNS).

The survival rate for persons diagnosed with brain tumors presently fluctuates around 34% and 36%. Individuals of a youthful demographic frequently demonstrate superior clinical outcomes. Nonetheless, early identification serves as the essential factor affecting the ultimate final result. The rapid identification of the illness facilitates the implementation of customized treatments. This early intervention markedly improves life expectancy. Magnetic Resonance Imaging (MRI) represents the preeminent diagnostic modality for this purpose. The MRI serves as a non-invasive diagnostic. It offers improved soft-tissue imaging without employing ionizing radiation. Clinicians generate diverse pulse sequences by modifying acquisition parameters. The parameters encompass the Time to Repetition (TR) and Time to Echo (TE). Commonly produced sequences encompass T1-weighted, T2-weighted, and Fluid-Attenuated Inversion Recovery (FLAIR) images. Although these sequences provide exhaustive anatomical detail, the resulting data volume can overwhelm even experienced radiologists. Consequently, automated diagnostic systems have transitioned from optional enhancements to clinical necessities. These technologies accelerate the diagnostic workflow. They also reduce human errors associated with clinical fatigue. However, significant obstacles remain due to the intrinsic variability of tumor structures. Lesions vary extensively in size and shape. Their anatomical locations also differ significantly. Furthermore, the analysis of complex textural features demands high clinical competence. This complexity necessitates the use of sophisticated computational models. Such models are required for precise classification across categories like Glioma, Meningioma, and Pituitary tumors.

Prior automation efforts depended on primitive neural networks. The latest developments in Transfer Learning and deep learning have contributed to higher accuracy levels. However, an important deficiency persists in existing diagnostic abilities. A number of conventional models demonstrate difficulties in generalizing whenever faced with images from various scanners or methods. The clinical translation is frequently hindered by hardware irregularities. Variations in scanner manufacturer and field strength produce inconsistent image qualities and “fuzzy” artifacts. In addition, biological heterogeneity implies that tumors often interact with healthy tissue lacking clear edges, leading to it challenging to organize them into categories. The proposed methodology is specifically designed to address these challenges through a multi-modal feature fusion pipeline.

The system begins with a preprocessing step which employs Guided Image Filtering (GIF) and normalization in order to make sure that the contrast remains consistent across all datasets^4^. This model expands beyond single-descriptor techniques by combining three distinct approaches: the Color Coherence Vector (CCV) for spatial connectivity, the Gray Level Co-occurrence Matrix (GLCM) for second-order texture, and the Local Ternary Pattern (LTP) for local intensity patterns. Specifically, the CCV implementation has been made possible by breaking down MRI intensity distributions into 27 different categories. This enables the system sort the pixels into coherent or incoherent categories based on the degree to which they connect in the surrounding space^5^. To handle the resulting multidimensional feature space and prevent the data from leaking, Principal Component Analysis (PCA) has been employed to reduce the feature vector. The transformation has a primary application on the original training data. In the end, the system ensures sure that learning patterns remain stable, by adding these new features to the PCA-DenseNet121 architecture. This method, that turns stronger by periodic dropouts and systematic data shuffling, seeks to reduce misclassification while functioning well with the wide range of images seen in contemporary healthcare settings^6^.

### Motivation of proposed work

The motivation for this proposed effort has arisen from the urgent clinical requirement for the diagnosis of early and precise brain tumors. These malignancies constitute approximately 90% of central nervous system neoplasms. It has shown an alarming death rate, having survival statistics ranging from 34 to 36%. The improved diagnostic procedures have helped substantially to improve patient outcomes and the rates of survival. The present work seeks to investigate a novel method for an earlier diagnosis and enhanced therapeutic approaches. The conventional manual analysis of magnetic resonance imaging (MRI) is becoming increasingly inadequate. This is due to the immense daily volume of scans and the inherent risk of human fatigue. Additionally, there has been a global lack of specialized radiologists to address these challenges. The integration of artificial intelligence and machine learning has been investigated. These technologies can analyze vast amounts of imaging data quickly and accurately. This has supported radiologists to arrive at better-informed judgments that improve patient care. In addition, a substantial variation in tumor form usually undermines diagnostic accuracy. The tumors have frequently exhibited irregular shapes, sizes, and placements. While the technical hurdles such as environmental effects and lighting variations further obfuscate clinical signals. The work effort is to investigate sophisticated image preprocessing techniques and resilient training datasets to improve the model reliability. The objective of this proposed work is to create a methodology that incorporates the various real-world settings in imaging. While the existing deep learning models show promise, they often struggle with generalization. The existing models have lacked sensitivity to subtle textural variations within complex backgrounds. Consequently, there has been an urgent requirement for advanced frameworks for the computational work. These frameworks have to give comprehensive image improvement. This specification encompasses automatic segmentation. Enhanced feature extraction serves as essential for the ultimate architecture. Such systems will assist healthcare professionals in achieving high detection rates with minimal error. Ultimately, these improvements facilitate timely intervention to improve patient survival outcomes.

### Contribution of research

This proposed work presents a PCA-DenseNet121 based deep learning model for automated brain tumor classification using MRI images that utilizes the fusion of features extracted from Gray Level Cooccurrence Matrix (GLCM), Local Ternary Pattern (LTP) and Color Coherence Vector (CCV). The key contributions include:

- The integration of DenseNet121 with PCA effectively reduces dimensionality while keeping important features for improving performance metrics of the proposed model.
- A systematic preprocessing approach such as image resizing, normalization, batch processing and label encoding which make sure efficient training and reliable performance.
- This research contributes to the field of transfer learning by showcasing how pre-trained model DenseNet121 can be effectively fine-tuned, dropout layers and data shuffling leading to consistent training and validation performance with low loss.

The rest of this research paper is presented in five different sections. Section “Related work” describes about the recent research work carried out this in this domain. Section “Proposed methodology” explains the methodology. Section “Experimental analysis” is dedicated to proposed work and algorithm. Section “Discussion” is result analysis and Section “Conclusion” for conclusion.

## Related work

Tumors, interstitial lung illnesses, heart disease, and tuberculosis are only a few examples of medical oddities. X-ray, magnetic resonance imaging (MRI), computed tomography (CT), positron emission tomography (PET), single photon emission computed tomography, or ultrasound scanning can be used to diagnose and predict the condition. To comprehend an image, it is necessary to look for abnormalities, identify their boundaries, and estimate the size and severity of those anomalies. Lack of human specialists, weariness, hefty consultation fees, and rough estimation techniques all restrict image understanding’s usefulness. In addition, the medical anomalies’ forms, locations, and structures vary widely. Even the most specialist doctors have difficulty in determining the cause of the problem. As a result, human professionals frequently feel the need for assistance in comprehending medical photographs precisely. This is the driving force behind the development of clever picture recognition systems. Suter, Y et al.^7^ in their paper has achieved by adopting a deep learning technique for performing regression task for medical images. The high-risk patients with brain tumor of severe grade were considered for the regression task. Despite the good results, the results were unstable because of the lack of sufficient dataset. Several radiomic features like shape, size, location etc. with support vector classifier resulted in good accuracy for BraTS 2018 survival prediction task.

The evaluation is done based on hold-out and repeated cross validation. Logic regression and ensemble SVC models have obtained best results compared to other techniques. 8. Li, Y., & Shen, L.^8^ have proposed deep learning architecture to deal the multimodal brain tumor segmentation challenge and prediction of survival of the diseased person. The tumors were segmented with different perspective by using Multi Branch Fully Convolutional Residual Networks (MBFCRN) and Multi-view deep learning framework (MvNet). SPNet was proposed for the survival prediction. Images of Glioblastoma and lower grade glioma from BraTS 17 dataset were considered by the author. The patients were classified into three groups based on their predicted survival period. With 55% of classification accuracy, it has won the fifth position in BraTS 17 competition. Tripathi, S. et al.^9^, has proposed an deep learning technique to crop the tumor region from the MRI image to help the medical practitioner to treat diseased area precisely. The encoders are connected interconnected in such a way that the feature maps are transfers to the consecutive layers without loss of information. Parametric rectified linear unit (PRELU) and cross channel Normalization (CCN) are adopted to achieve better test results for the images from the cancer imaging archive data set.

The metric like mean intersection over union, class average accuracy, and global accuracy were calculated to validate the performance of the algorithm. Amin, J. et al.^10^ have analyzed human brain images to detect the abnormalities using intelligence of machine. The images from the MICCAI, BraTS challenge datasets were preprocessed before further analysis. Partial Differential Diffusion Filter (PDDF) is used to enhance the region of interest from the input image and Otsu thresholding technique is adopted for segmenting the tumor region. The classifiers are fed with individual features like Local Ternary Pattern (LTP), GLCM features and finally with combination of LTP and GLCM features. Out of several classifiers tested, k-Nearest Neighbor (KNN) produced better accuracies for BraTS 2012, 2013 & 2015 challenge datasets.Arunachalam, M., & Savarimuthu, S. R.^11^ have proposed an algorithm to segment the necrosis from the normal cells in the tumour tissue. T1 weighted image from BRATS challenge was considered for evaluating the performance of the algorithm. The contrast of the input image is enhanced by a No subsampled Shear let Transform (NSST) and fed to the adaptive contextual clustering technique to classify the pixels as white matter, cerebrospinal fluid and gray matter.

The performance of the method was evaluated with various quality metrics like DICE coefficient, overlap fraction, extra fraction and Jaccard index (JAC). Al-Hadidi, M. R. et al.^12^ have proposed an CNN architecture to glioma tumor from the MRI of brain. The challenge in this task is that glioma tumor gets diffused with the neighboring tissue and it is location shape and size vary immensely. The images from cancer imaging dataset were used to test the performance of the algorithm. The contrast of the input images is enhanced in the pre-processing stage. Further the super pixel segmentation is performed using Simple Linear Iterative Clustering (SLIC) procedure. The labeled subarea is used to train the CNN network for further classification. Overall, 75% testing accuracy was obtained for the dataset. Sharif, M. I et al.^13^ have proposed a deep learning-based support system for multi modal brain tumor classification problem. The experimentation was carried out using BRATS 2018 and 2019 challenge dataset. Precise classification was achieved with entropy- kurtosis based high feature values and metaheuristics based modified genetic algorithm^14^.

The features were fed to several classifiers for further classification. The linear discriminant classifier performed the classification at faster pace but, cubic SVM classifier even though took a longer time for computation resulted in good classification accuracy^15^. In the beginning, the method uses a minimum spanning tree graph-theoretic approach to lower the fraction of samples in the dataset that are erroneously labeled, based on samples obtained from prior tissue probability maps^16^. After that, a supervised KNN classifier is utilized to categorize the complete 3D image using the rectified set of samples^17^. An MRI dataset from the Alzheimer’s disease Neuroimaging Initiative (ADNI) and an MRI dataset from the Open Access Series of Imaging Studies were both used in this investigation. This methdlogy used a combination of shear let-based descriptors and deep features to classify AD^18^.

## Proposed methodology

**Fig. 1:**
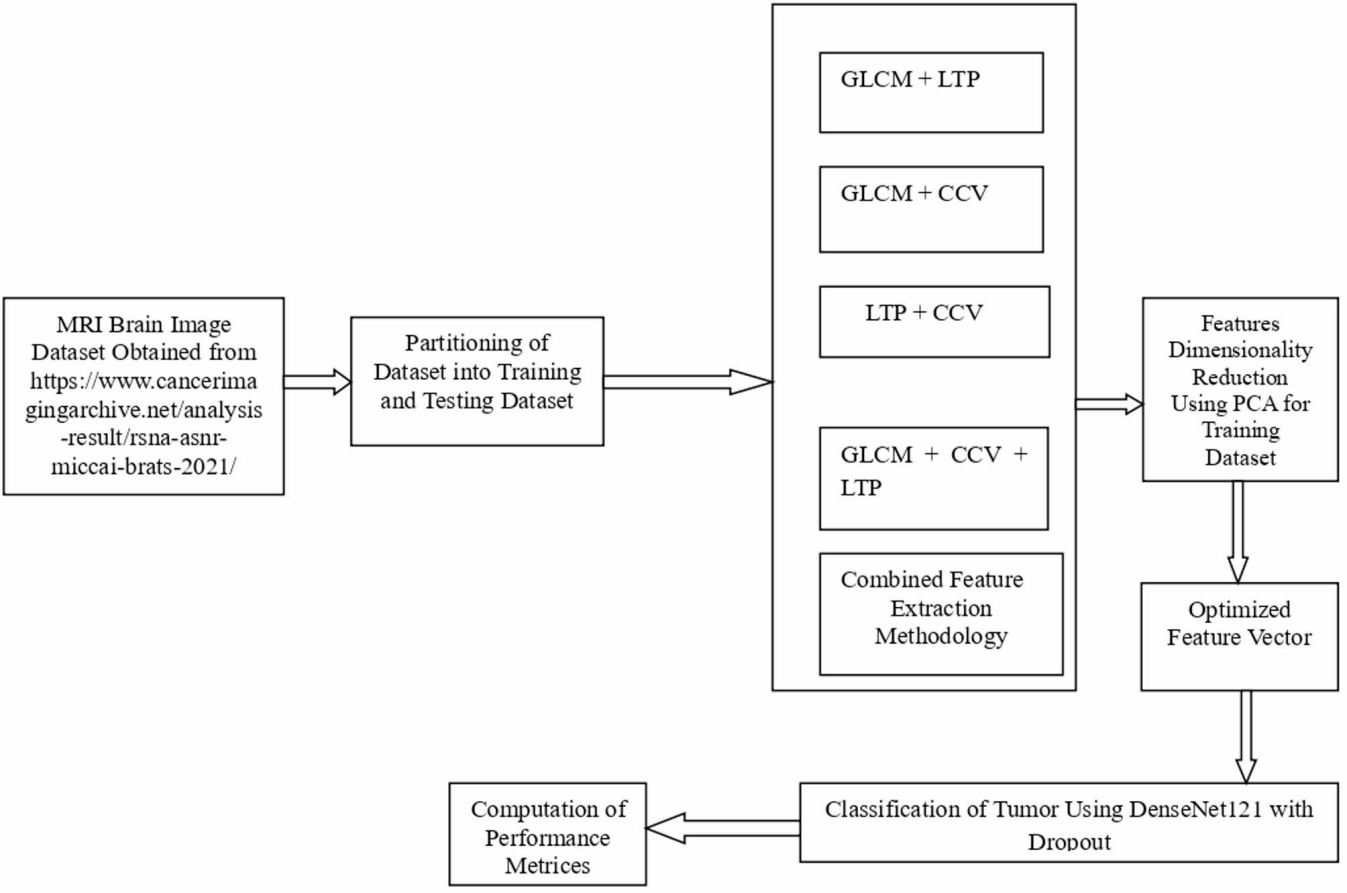
Work flow of proposed Brain Tumor identification process in MRI Image ^49^.

The identification of brain tumors is a complex task compared to standard retrieval methods^19^. This complexity arises from the intricate nature of tumor boundaries. Furthermore, MRI scans exhibit high variability in appearance^20^. Malignant cells may manifest as opaque regions against light backgrounds or vice versa^21^. Several challenges necessitate a robust classification approach^22^. These include environmental artifacts, lighting variances, and viewpoint abnormalities^23^. Such an approach is required to achieve high detection rates with minimal error. The proposed methodology begins with the acquisition of MRI brain image data from the BraTS 2021 dataset^27^, as illustrated in Fig. 1. All images are resized to 128 × 128 pixels to ensure compatibility with the DenseNet121 architecture. The data is then subjected to normalization and scaling^24^. These steps align input distributions to ensure better model convergence^25,26^. Image enhancement is achieved through Guided Image Filtering (GIF)^28^. This method offers superior edge preservation compared to bilateral filters. It also provides lower computational overhead. Following enhancement, the K-Means clustering algorithm is utilized for automated segmentation^29^. This process isolates the Region of Interest (ROI) from surrounding healthy brain tissues. K-Means is an unsupervised learning technique. It has been dividing the image into k separate clusters. This can be achieved by iteratively decreasing the squared Euclidean distance between pixel intensity values and the corresponding cluster centroids. The procedure has been described by the following objective function given by Eq. (1).

1 $$J = \mathop \sum \limits_{j = 1}^{kc} \mathop \sum \limits_{i = 1}^{n} x_{i}^{\left( j \right)} - ct_{j}^{2}$$

where $x_{i}^{\left( j \right)} - ct_{j}$ is the chosen distance measure between a pixel ${x}_{i}$ and the cluster center ${ct}_{j}$. To provide a comprehensive technical overview of the algorithmic steps, the following pseudocode outlines the implementation of K-Means Clustering for segmentation:

**Algorithm 1:**
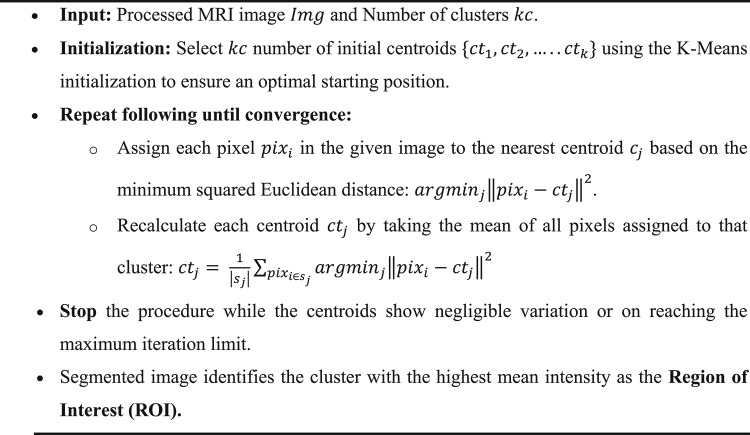
K Means Clustering for Segmentation of Image

Once the ROI is isolated, the dataset is partitioned into independent training and testing subsets. This process uses a strict patient-level split. Such a measure is critical to prevent data leakage. It ensures that slices from the same subject do not appear in both sets. This avoids artificially inflated performance metrics. Without this split, the model might recognize specific patient anatomy instead of actual pathological features^30^. Following partitioning, a combined feature extraction methodology is applied. This captures intricate textural and spatial characteristics from the segmented regions. The process utilizes the Gray-Level Co-occurrence Matrix (GLCM)^19^. It also incorporates Local Ternary Patterns (LTP) and the Color Coherence Vector (CCV)^31^. These descriptors are evaluated in various fusion combinations. These include GLCM + LTP, GLCM + CCV, and LTP + CCV. A comprehensive GLCM + CCV + LTP tri-fusion is also tested^21^. This evaluation identifies the most discriminative representation for the tumor. The fused data often exhibits high dimensionality^32^. To manage this, Principal Component Analysis (PCA) is used to reduce the feature vector. This reduction follows a specific mathematical procedure to maintain data integrity.

**Algorithm 2:**
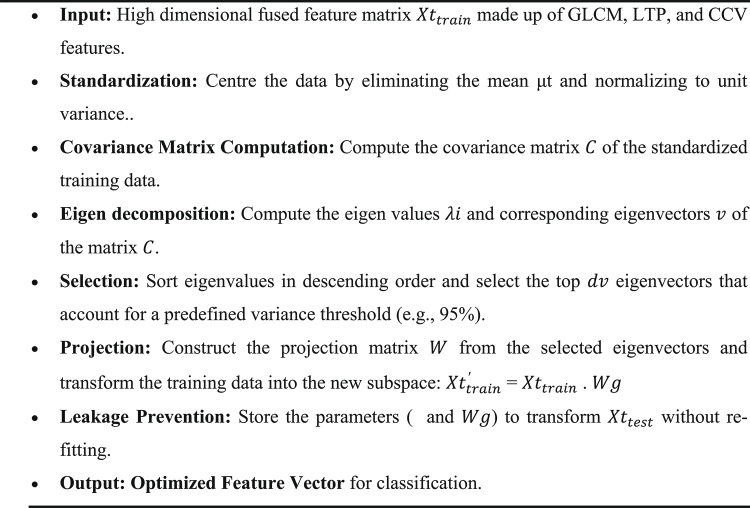
PCA-based Feature Optimization

The methodology integrates these algorithms to refine the model inputs. Using K-Means ensures the inputs are spatially relevant. Simultaneously, PCA ensures the features are statistically significant. To maintain scientific integrity, the PCA transformation is fitted solely on the training data. The scaling parameters have been derived only from this portion of the data. The chosen settings are subsequently applied to the testing set in an unilateral manner. This implies that the test information goes completely unobserved during the optimization procedure. The optimized vector had been evaluated employing a DenseNet121 model. This model incorporates dropout layers to mitigate overfitting. The process ends with the computation of performance measurements^33^. These metrics assess the diagnostic accuracy and reliability of the system. The last part emphasizes deep learning classification with an enhanced DenseNet121 architecture. This architecture is characterized by a dense connectivity pattern. In this pattern, each layer receives feature maps from all preceding layers as input. This design is particularly advantageous for medical imaging^34^. It alleviates the vanishing-gradient problem and strengthens feature propagation. This substantially reduces the number of parameters in comparison to conventional convolutional networks^35^. The Optimized Feature Vector (OFV) derived from the PCA procedure has been integrated into the classification head within this framework. The custom dropout layers have been deliberately utilized to inhibit the model from retaining training data^36^. Dropout has a method of regularization. It arbitrarily ignores chosen neurons during the training process. This inhibits excessive mutual adaptation for neurons. As a consequence, the network acquires stronger and redundant representations of tumor characteristics^37^. A dropout percentage of 50% (0.5) has been used for this investigation. This layer is set up prior to the ultimate completely connected layer. This implies that the model does not become excessively dependent on any singular feature component. This increases the ability of the model to generalize to the unobserved testing data^38^. The training procedure employs the Adam optimizer. A categorical cross-entropy loss function had been employed to optimize the values of the weights. The integration of K-Means, the PCA, and DenseNet121 provides a multiple layered safety system. This safeguard prevents against excessive underfitting and overfitting. The models efficiency is rigorously evaluated using the testing set^39^. This offers a validated assessment of the methods diagnostic accuracy.

### Region based clustering

**Fig. 2:**
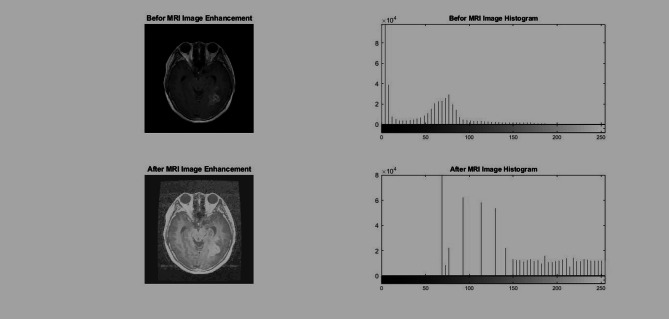
Input image with Histogram and Histogram equalization and its histogram of Glioma Tumor.

Region-based clustering is used to streamline the MRI image region, enabling improved understanding, and sufficiently precise analysis of features ensures that the machine complexity reduces during the process of analysis. A number of numerous segmentation methods are readily available, including thresholding, morphology-based, K-means, the watershed algorithm, and the cluster approach, as seen Fig. 2. The performance values for parameters such as PSNR (Peak Signal to Noise Ratio) and RMSE (Root Mean Square Error) have been determined for different methods and reported in Table 1. These evaluations have confirmed the fidelity of segmentation during the preprocessing phase. These values do not serve as direct inputs to the DenseNet121 classifier. However, they remain critical for assessing the K-Means algorithm. Effective isolation of the Region of Interest (ROI) requires minimal information loss. In this situation, a high PSNR and low RMSE indicate successful outcomes. These scores have been ensured that the delineated tumor region preserves critical textural and spatial features. These qualities have significance for further feature extraction using GLCM, LTP, and the CCV for further analysis. This process ensures high-fidelity segmentation. It is possible to prevent the emergence of substantial artifacts. This safeguard assures the integrity of the feature vector’s quality. The proposed approach has been quantified for clustering performance to ensure the optimized feature vector accurately represents diseased tissue^40^. This quality-control measure directly affects the ultimate classification accuracy and also explicitly indicates that this evaluation represents an essential bottleneck before feature extraction^41^. The performance of the K-Means segmentation is quantitatively assessed using PSNR and RMSE to ensure that the isolated ROI preserves the high-fidelity textural details necessary for subsequent feature fusion and deep learning classification^42^.

**Table 1:** PSNR and RMSE scores for different clustering method.

Region Based Clustering Method	PSNR	RMSE
Threshold Based	27.52	0.25124
Edge based	33.25	0.27456
Region Growing	35.54	0.26765
Proposed method	38.39	0.23436

### Feature extraction method: GLCM

**Fig. 3:**
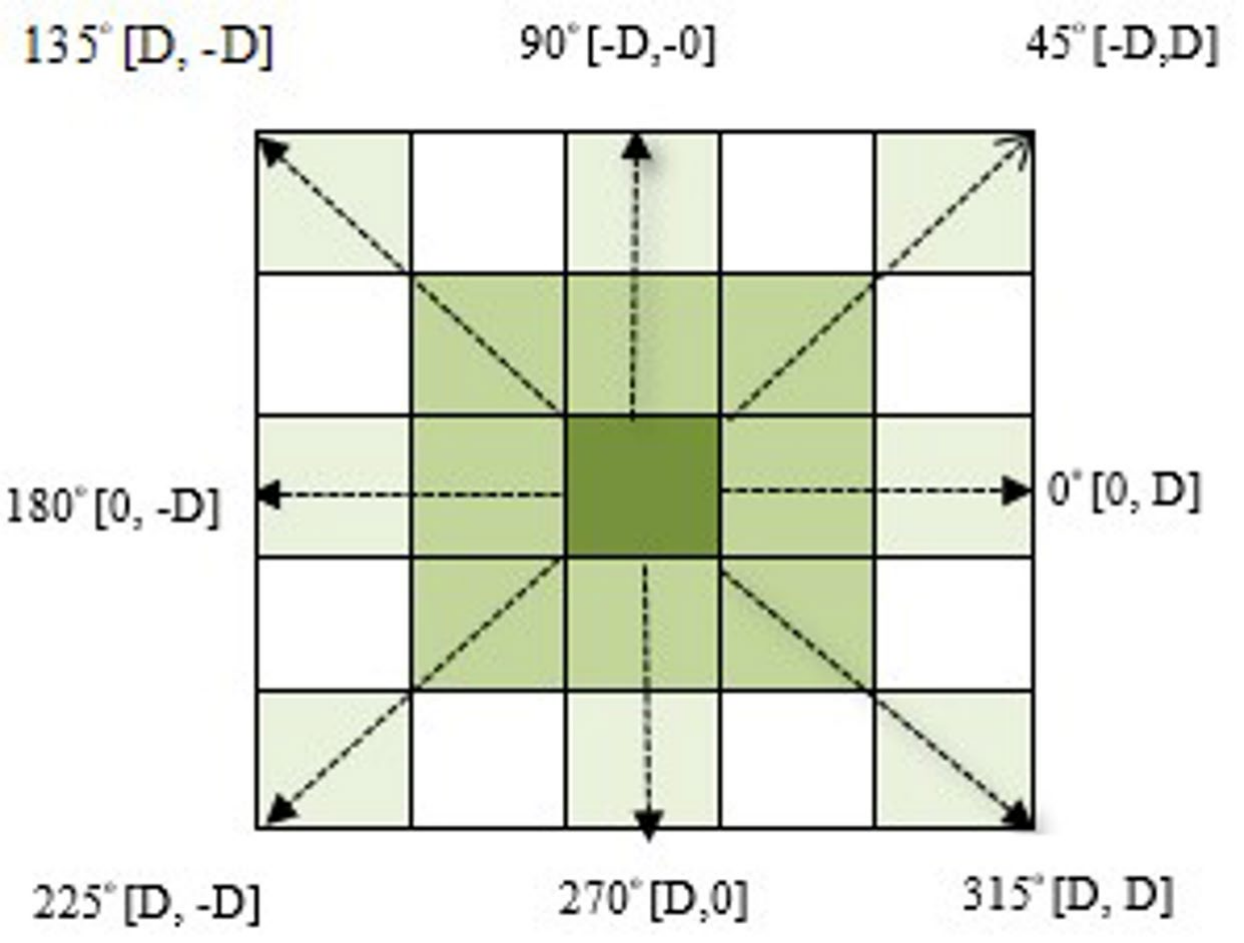
Cooccurrence Matrix.

The Gray Level Co-occurrence Matrix comprises a square matrix constructed from an input image. The size of the GLCM matrix corresponds to the total quantity of gray levels in the image being processed. As an illustration, an 8-bit image will include 256 Gy levels that vary from 0 to 255. For such an image, the GLCM matrix will consist of 256 rows by 256 columns, and every row/column will indicate one of the values of intensity values. The second-order statistics are calculated by considering a set of pixels that have been connected to one another in positive three-dimensional space. Gray Level Co-occurrence matrices offer unique mathematical facts about texture^43^. The GLCM matrix of an image is determined by the orientation and offset elements. The direction can be any of the eight potential directions, as shown in Fig. 3. The offset indicates the distance that exists between pixels. If the difference in distance between picture elements is one, the nearest neighboring picture element in the direction has been taken into account. This method allows for the extraction of many GLCM matrices from a single image.

#### GLCM features extraction

Let $f\left(u,v\right)$ characterises the standardized matrix, with N number of grey levels ${\mu }_{u} ,{\sigma }_{u} and {\mu }_{v}, {\sigma }_{yv}$ are mean and standard deviation of the bordering probability matrix ${P}_{u}\left(u\right)$ and ${P}_{v}\left(v\right)$ respectively as given by Eq. (2) to (3).

2 $$P_{u} \left( u \right) = \mathop \sum \limits_{n = 0}^{N - 1} g\left( {x,y} \right)$$

3 $$P_{v} \left( v \right) = \mathop \sum \limits_{m = 0}^{N - 1} g\left( {x,y} \right)$$

Mean of the marginal probability matrix ${P}_{u}\left(u\right)$ and ${P}_{v}\left(v\right)$ are given as Eqs. (4), (5), (6) and (7)

4 $$\mu_{u} = \mathop \sum \limits_{m = 0}^{N - 1} u\mathop \sum \limits_{n = 0}^{N - 1} f\left( {u,v} \right)$$

5 $$\mu_{u} = \mathop \sum \limits_{n = 0}^{N - 1} u P_{u} \left( u \right)$$

6 $$\mu_{v} = \mathop \sum \limits_{y = 0}^{N - 1} v\mathop \sum \limits_{x = 0}^{N - 1} f\left( {u,v} \right)$$

7 $$\mu_{v} = \mathop \sum \limits_{n = 0}^{N - 1} vP_{v} \left( u \right)$$

Standard deviation of the marginal probability matrix ${P}_{u}\left(u\right)$ and ${P}_{v}\left(v\right)$ are given as an Eqs. (8), (9), (10) and (11)

8 $$\sigma_{u}^{2} = \mathop \sum \limits_{u = 0}^{N - 1} \left( {u - \mu_{u} } \right)^{2} \mathop \sum \limits_{v = 0}^{N - 1} f\left( {u,v} \right)$$

9 $$\sigma_{v}^{2} = \mathop \sum \limits_{v = 0}^{N - 1} \left( {u - \mu_{v} } \right)^{2} \mathop \sum \limits_{u = 0}^{N - 1} f\left( {u,v} \right)$$

10 $$P_{u + v} \left( l \right) = \mathop \sum \limits_{u = 0}^{N - 1} \mathop \sum \limits_{v = 0}^{N - 1} f\left( {u,v} \right) where l = u + v:for l = 0 to 2\left( {N - 1} \right)$$

11 $$P_{u - v} \left( l \right) = \mathop \sum \limits_{u = 0}^{N - 1} \mathop \sum \limits_{v = 0}^{N - 1} f\left( {u,v} \right) wherel = u - v: for l = 0 to 2\left( {N - 1} \right)$$

#### Energy

The energy (E) is calculated as the total number of squares of each element in the GLCM matrix. It gives a value in the range of 0 to 1. An energy level of one indicates the image is consistent in value. It also indicates the homogeneity of the image using Eq. (12).

12 $$P_{u - v} \left( l \right) = \mathop \sum \limits_{u = 0}^{N - 1} \mathop \sum \limits_{v = 0}^{N - 1} f\left( {u,v} \right)^{2}$$

#### Contrast

Contrast evaluates an image’s spatial frequency in addition to the distinct moments of the GLCM. It represents the distinction across the highest and lowest values of a neighboring collection of pixels. For an unchanging image, contrast equals zero. Inertia and variance refer to identical quality. It’s also known as inertia. Contrast can be determined from $f\left(u,v\right)$ utilizing the following Eq. (13).

13 $$Contrast\left( C \right) = \mathop \sum \limits_{m = 0}^{N - 1} \mathop \sum \limits_{n = 0}^{N - 1} \left( {u - v} \right)^{2} f\left( {u,v} \right)$$

#### Correlation

The input image f (u, v) has been extremely correlated across the nearest pixels; subsequently, it might state the image has been correlated automatically (the autocorrelation of input information in itself is meant to follow changing a single pixel). The coefficient of correlation, which may be computed using the Eq. (14), quantifies the linear dependency among the number of pixels at each point.

14 $$Corr = \mathop \sum \limits_{m = 0}^{N - 1} \mathop \sum \limits_{n = 0}^{N - 1} \frac{{\left( {x \times y} \right)f\left( {x,y} \right) - \left( {\mu_{x} \times \mu_{y} } \right)}}{{\left( {\sigma_{x} \times \sigma_{y} } \right)}}$$

#### Homogeneity

Homogeneity represents a uniformity in the setup of an input image f (u, v). An input image has been deemed homogenous provided its layout adheres to a consistent standard. An image with a homogeneity value of 1 serves as a constant. The following Eq. (15) is one way to express it numerically.

15 $$Homogeneity\;\left( H \right) = \;\mathop \sum \limits_{m = 0}^{N - 1} \mathop \sum \limits_{n = 0}^{N - 1} \frac{{f\left( {u, v} \right)}}{{1 + \left| {u - v} \right|^{2} }}$$

### Feature extraction using local ternary pattern (LTP)

The Local Ternary Pattern (LTP) has become an advancement of the conventional Local Binary Pattern (LBP). It has been developed to enhance robustness and increase feature discrimination in environments with excessive noise. The LBP has a straightforward binary threshold. The LTP has a ternary encoding method. This technique operates by establishing a precise threshold width surrounding the pixel at the centre. Equation (16) divides the categorization of neighbouring pixels into three separate values: 1, 0, or −1. The values have been determined by the intensity of neighbouring pixels in relation to the central pixel and the one that is chosen threshold.

16 $${\mathrm{LTP}}\;\left( {p, a, t} \right) = \left\{ {\begin{array}{*{20}c} {1, p \ge a + t} \\ {0, \left| {p - a} \right| < t} \\ { - 1 p \le a - t} \\ \end{array} } \right.$$

Following this thresholding process, the adjacent pixels are organized into a ternary arrangement. Because a raw histogram of these ternary values would yield an excessively broad range, the pattern is split into two separate binary patterns: the upper and lower patterns. These binary strings are then converted into histograms and concatenated. The resulting LTP descriptor provides a more detailed representation of the image texture than LBP, though it is twice the size in terms of dimensionality.

### Feature extraction using color coherence vector (CCV)

The proposed methodology follows a strict pipeline designed to prevent data leakage by ensuring that the training and validation sets are isolated before any feature optimization occurs^44^. First, the raw MRI image dataset is partitioned into a training set (X_train_, 80%) and a validation set (X_val_, 20%). All subsequent preprocessing and feature extraction steps—including K-Means clustering for segmentation and the calculation of Color Coherence Vectors (CCV), Gray Level Co-occurrence Matrix (GLCM), and Local Ternary Patterns (LTP) are performed within this split framework. Specifically, the CCV process quantizes the color space into 27 hues and classifies pixels as coherent or incoherent based on a connectivity threshold t set at 1% of the image size^45^. After extracting these hybrid features, Principal Component Analysis (PCA) is employed to reduce dimensionality. Crucially, the PCA model is fitted only on the training features to identify the principal components. These learned parameters are then used to transform the validation features^46^. This ensures that the validation data remains entirely unseen during the optimization phase, providing an unbiased evaluation of the model’s performance^47^. Through batch-wise forward and back-propagation, the model modifies parameters based only on the training distribution, with validation carried out at the end of each epoch to ensure generalizability.

The proposed approach incorporates an extensive feature fusion strategy. Descriptors have been obtained from the Gray-Level Co-occurrence Matrix (GLCM), Local Ternary Pattern (LTP), and Color Coherence Vector (CCV). The descriptors have been combined into a singular blended feature vector. The aforementioned ensures the model comprehensively incorporates both global spatial distributions and local textural variations. The LTP descriptor alone provides a dual-histogram representation. This representation is twice the size of a standard LBP. A systematic scaling approach is employed to prevent features with varying ranges from disproportionately influencing the learning process. The input images have undergone to initial normalization to conform to DenseNet121 standards. The integrated feature matrix has been normalized before doing Principal Component Analysis (PCA). This phase ensures dependable reduction of dimensionality. To maintain scientific integrity and prevent data leakage, a forward only scaling framework is applied. The dataset is partitioned into an 80% training and 20% validation split. The PCA model and standardization parameters are fitted exclusively on the training features. These parameters specifically include the mean and variance. It has to be used to modify the validation set. This stringent methodology ensures that the validation information will remain completely unobserved during the optimization process^48^. Consequently, it has preserved the integrity of the performance metrics and ensured that all attributes contribute equally to the ultimate classification.

## Experimental analysis

The PCA-DenseNet121-based convolutional neural network model was executed on a machine that utilized an Intel(R) Core (TM) i7-8565U CPU configured at 1.80 GHz to 1.99 GHz and 8 GB of RAM. TensorFlow (version 2.3.0) and Keras (version 2.4.3) have been two open-source libraries widely utilized in Google’s Colab framework for development. TensorFlow is a Python library that may be deployed to create models that use deep learning, whereas Keras includes a Python deep learning API. The initial hyperparameters for the analysis include an initial learning rate of 0.001 to maintain training stability and a batch size of 64 to optimize gradient estimation and computational efficiency. A dropout rate of 0.5 was strategically integrated into the fully connected layers to mitigate overfitting by promoting redundant feature representation, which is critical for interpreting characters obscured by medical artifacts. The training process utilized a validation patience of 5 for early stopping to prevent the model from memorizing training noise, thereby ensuring maximum generalization. The implied approach’s outcomes appear to be appropriate for a population size of 20. Above 20 population sizes lead to longer process times with a small improvement in performance. As a result, every experiment requires a population of 20. Moreover, if the population grows beyond 20, the effectiveness of the proposed approach measures decreases. The specified dataset’s information will be provided in the subsequent subsections.

### Dataset description

The set of data is perfect for demonstrating the effectiveness of the proposed approach since it possesses a lot of variety in dimensions, position, hue, and arrangement, lighting, and all of these factors have been identified by experts as among the most difficult and prominent datasets. Thus, an overall of roughly 7023 MRI images^49^ is employed for this indicated methodology, which are separated into four distinct categories: pituitary tumor, glioma tumor, meningioma tumor, and normal classes (no tumor). Out of these 7023 images, 5619 are chosen for training; tenfold cross-validation is then used to increase the model’s performance during training. The ten-fold approach was used to divide the standard MRI brain tumor images that were obtained and showed data about patients into ten distinct groups. While every round of training of a model uses nine subsets as the training set, the test data also contains the residual segment. To reduce the likelihood of prejudice, a significant amount of data that is accessible is used to train models across ten iterations. Furthermore, the model weights of the convolutional layers are continuously adjusted after every iteration, thus improving the learning process’ efficacy^50^. In contrast, 1404 images are taken for the testing procedure, as shown in Table 2. During the implementation of the proposed approach, it became apparent that if the total number of images is not significant, it might end up in reduced accuracy of models and overfitting. To prevent these kinds of problems, the proposed approach employs customary methods for data augmentation to enhance the overall size of the dataset. The data augmentation techniques have significance for datasets with few visuals because these methods improve not simply the amount of the dataset but also the variety of input visuals, enabling the model that was developed to be more adaptable with complex backgrounds and boosting the model’s resilience^51^.

**Table 2:** Breakdown of data types within the dataset.

Data	Training Dataset (80%)	Testing Dataset (20%)	Total Dataset (100%)
Glioma	1297	324	1621
Meningioma	1316	329	1645
Pituitary	1406	351	1757
No Tumor	1600	400	2000
Total	5619	1404	7023

### Evaluation metrics

**Fig. 4:**
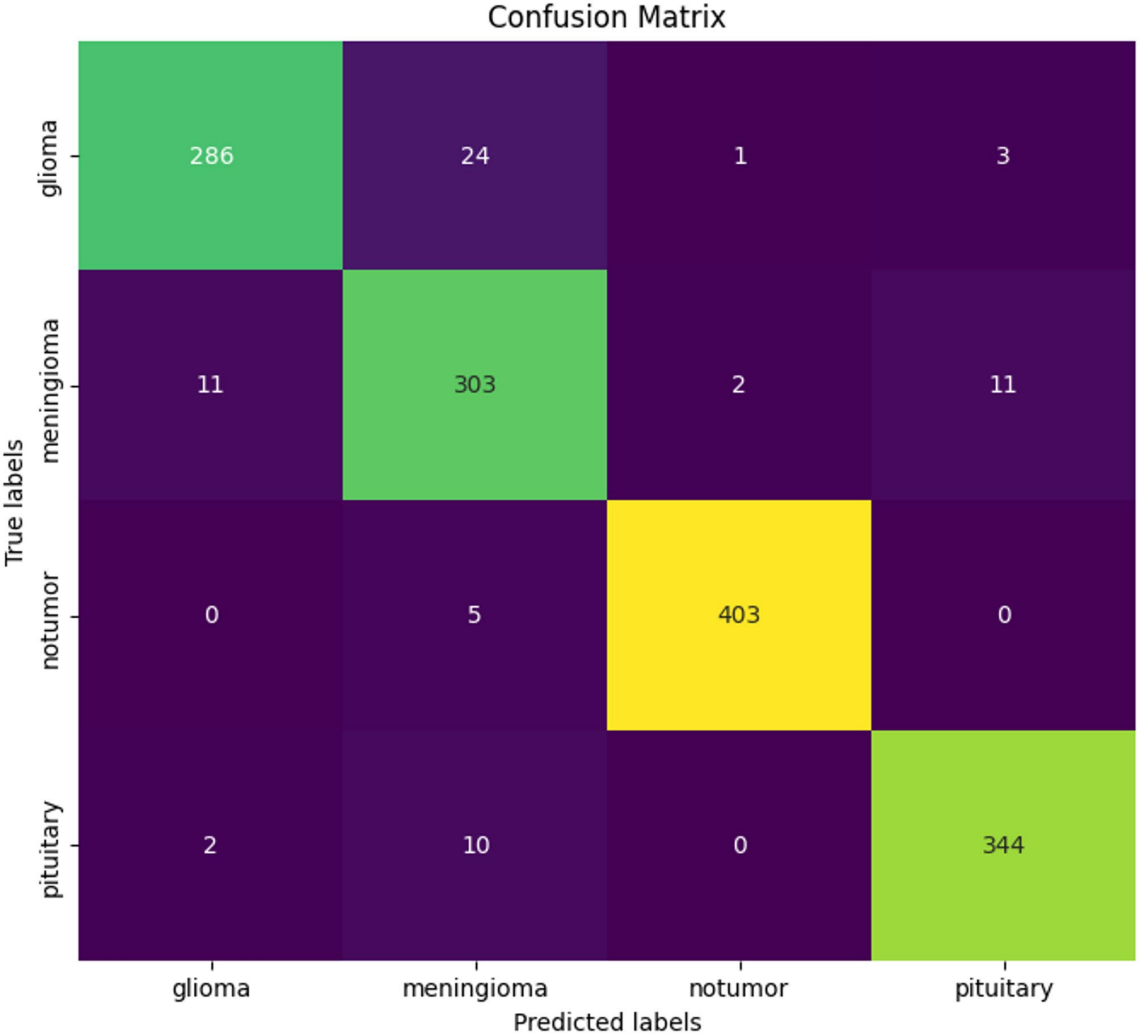
Confusion Matrix for the DenseNet121 Classification Model.

The efficacy of the PCA-DenseNet121-based convolutional neural network model, which incorporates three feature extraction methodologies such as Gray Level Co-Occurrence Matrix (GLCM), Local Ternary Pattern (LTP), and Color Coherence Vector (CCV) in classifying brain images into four categories like glioma, meningioma, no tumor, and pituitary was assessed. Table 3, Table 4, Table 5, Table 6, Table 7, Table 8 demonstrate that the performance of GLCM + LTP + CCV techniques produced better true positives than individual feature extraction methodology, which means that the classification rate^52^ has been improved when CCV and LTP features have been combined with GLCM features. As the threshold for LTP is between 0 and 1 for LTP, it makes it easy to combine with CCV features. The collected features are fed into the PCA-DenseNet121-based convolutional neural network model, which performs all of the model’s training, testing, and assessments. The confusion matrix shown in Fig. 4 has quantitatively illustrated the classification efficacy of the proposed PCA-DenseNet121 methodology. This methodology has incorporated a tri-fusion feature extraction technique comprising GLCM, LTP, and CCV. The matrix shows the model has attained an elevated accuracy for the diagnosis in all of the four categories. The proposed approach has been able to accurately identify the 96 cases of Glioma, 95 cases of Meningioma, 51 cases of No Tumor, and the 124 cases of Pituitary tumors.

**Table 3:**
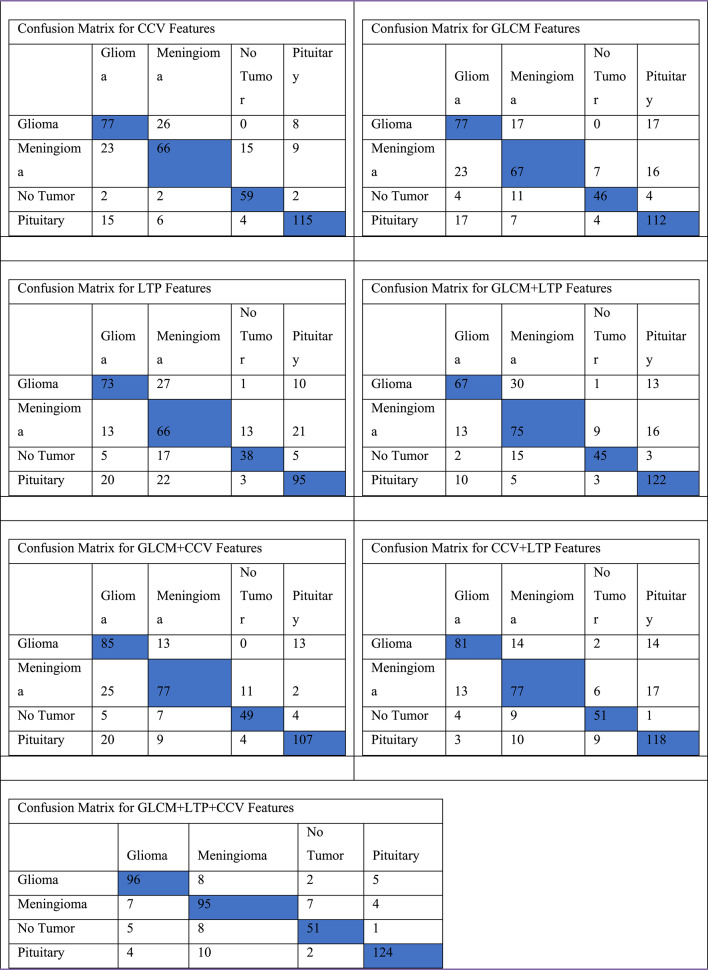
Confusion matrix for all the combination of features techniques for proposed work.

**Table 4:** Performance evaluation of GLCM feature extraction.

Class No	Accuracy	Precision	F1 score	Sensitivity	Specificity
1	80.71	62.96	0.69	76.58	82.33
2	82.60	72.64	0.70	66.96	89.26
3	91.12	76.56	0.76	75.38	94.72
4	85.95	84.92	0.80	76.43	91.74

**Table 5:** Performance Evaluation of LTP Features Extraction.

Class No	Accuracy	Precision	F1 score	Sensitivity	Specificity
1	78.16	65.77	0.66	65.77	83.97
2	70.65	50.00	0.54	58.41	75.74
3	86.08	69.09	0.63	58.46	93.23
4	77.05	72.52	0.70	67.86	83.10

**Table 6:** Performance Evaluation of GLCM + CCV Features Extraction.

Class No	Accuracy	Precision	F1 score	Sensitivity	Specificity
1	92.19	85.71	0.86	86.49	94.41
2	89.27	78.51	0.81	84.07	91.25
3	93.61	82.26	0.80	78.46	96.63
4	93.37	92.54	0.91	88.57	96.03

**Table 7:** Performance Evaluation of GLCM + LTP Features Extraction.

Class No	Accuracy	Precision	F1 score	Sensitivity	Specificity
1	81.75	72.83	0.66	60.36	90.64
2	77.83	60.00	0.63	66.37	82.39
3	90.35	77.59	0.73	69.23	95.31
4	86.07	79.22	0.83	87.14	85.39

**Table 8:** Performance Evaluation of GLCM + LTP + CCV Features Extraction.

Class No	Accuracy	Precision	F1 score	Sensitivity	Specificity
1	92.19	85.71	0.86	86.49	94.41
2	89.27	78.51	0.81	84.07	91.25
3	93.61	82.26	0.80	78.46	96.63
4	93.37	92.54	0.91	88.57	96.03

The Pituitary class has demonstrated the greatest classification success as comparison to all other groupings. The minor misclassifications have predominantly happened between the classification of the Glioma and meningioma classes. These inaccuracies have been attributed to the common intricate textural borders between these different kinds of tumors. The findings confirm the effectiveness of integrating deep features^53^ with conventional texture descriptors. Moreover, the tri-fusion methodology has produced markedly superior true positive rates in comparison to the singular feature extraction approaches. Subsequently, 344 of 356 pituitary cases had been correctly recognized, with two classified incorrectly as gliomas and ten as meningiomas. These outcomes indicate that the model offers outstanding performance in classification^54^, especially when it comes to accurately categorizing tumor instances and then testing the efficiency of the proposed approach utilizing accuracy, recall, precision, and F1 score metrics using a confusion matrix, and the Eqs. (17), (18), (19) and (20) are included below.

17 $${\mathrm{Accuracy}} = \frac{{{\mathrm{True}} {\mathrm{Positive}} + {\mathrm{True}} {\mathrm{Negative}}}}{{{\mathrm{Total}} {\mathrm{number}} {\mathrm{of}} {\mathrm{Samples}}}}$$

18 $${\mathrm{Recall}} = \frac{{{\mathrm{True}} {\mathrm{Negative}}}}{{{\mathrm{Trun}} {\mathrm{Negative}} + {\mathrm{False}} {\mathrm{Positive}}}}$$

19 $${\mathrm{Precision}} = \frac{{{\mathrm{True}} {\mathrm{Postive}}}}{{{\mathrm{True}} {\mathrm{Postive}} + {\mathrm{False}} {\mathrm{Positive}}}}$$

20 $$F1 {\mathrm{Scor}}e = 2*\frac{{{\mathrm{Precision}}*{\mathrm{recall}}}}{{{\mathrm{Precision}} + {\mathrm{recall}}}}$$

Overall, a model accurately classified brain tumor subtypes with only a few mistakes, resulting in an appealing method for automated brain tumor classifications.

**Fig. 5:**
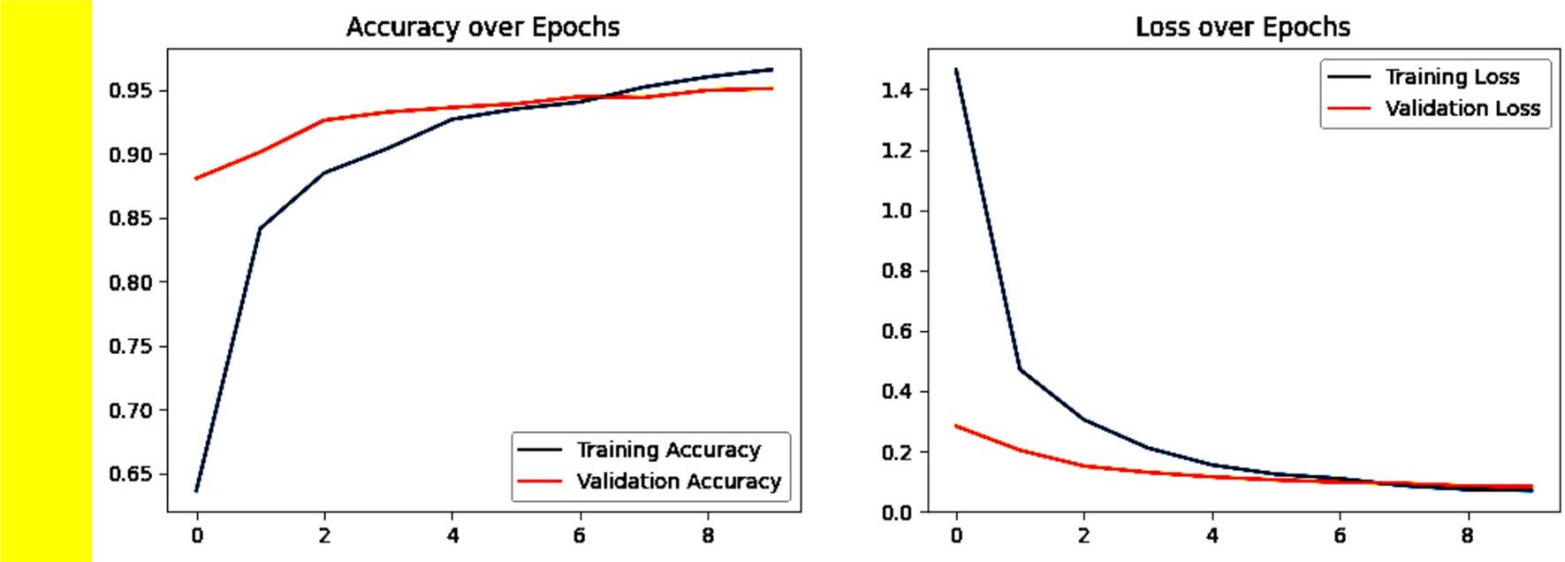
(**a**) Accuracy over Epochs. (**b**) Loss over Epochs.

The model’s process of learning during training is visibly illustrated in two plot. The accuracy trend can be observed in Fig. 5a, whereby the initial training accuracy shown by blue line starts at around 65%, rises steeply, and eventually exceeds 95.89% by the completion of the epoch. Similarly, the measurement of validation accuracy shown by red line begins at 87%, gradually increases, and roughly matches with the training accuracy as the epochs advance, eventually maintaining approximately 95.89%.

Figure 5b shows the loss values throughout the training. The training loss shown by blue line starts at relatively high value of 1.4, decreasing in the initial epochs before gradually approaching near zero as training progresses. The validation loss shown by red line follows a similar downwards trend but remains slightly higher than the training loss, which shows the minimal overfitting. Overall, the graphs show that the minimum gap between training and validation accuracy as well as the decreasing the loss tends.

**Fig. 6:**
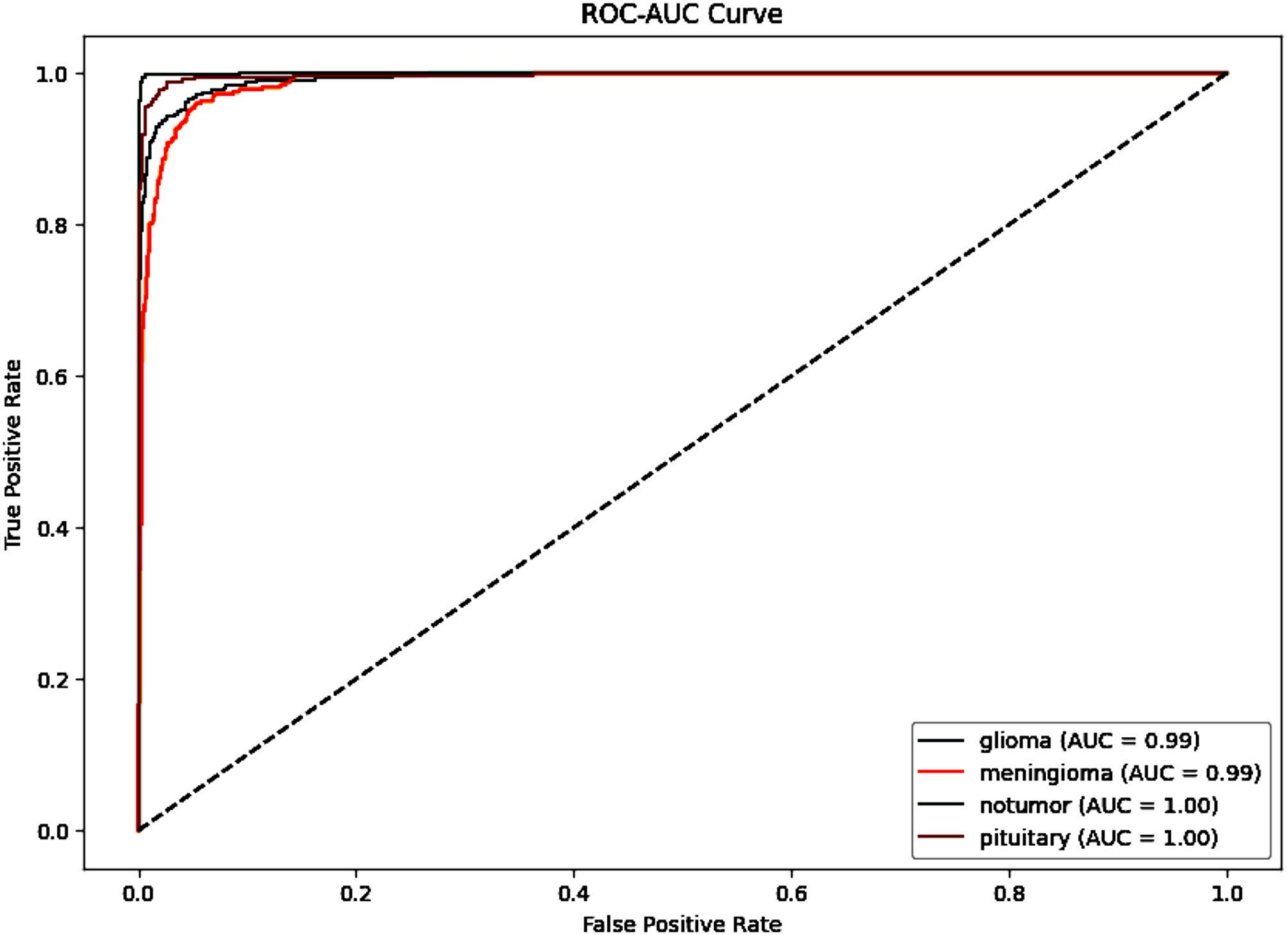
ROC curve for the DenseNet121 Classification Model.

As shown in Fig. 6, the ROC-AUC curve shows the model’s ability to differentiate between brain tumor types: glioma, meningioma, nontumor and pituitary. The curve plots the True Positive Rate (TPR) against the False Positive Rate (FPR), giving a classification performance of the model. The Area Under the Curve (AUC) values show near-perfect discrimination with glioma and meningioma achieving 0.99 AUC, while nontumor and pituitary reach a flawless 1.00 AUC. The curves are clustered near the top-left corner, signifying a high true positive rate with minimal false positives across all classes. The nearly perfect AUC scores confirm the model’s accuracy and reliability, particularly in distinguishing nontumor and pituitary cases. Table 9 provides a comparison of performance with earlier reported studies.

**Table 9:** Comparative Evaluation of MRI Image Analysis Models for Brain Tumor Detection.

Authors	Dataset	Model	Performance (%)
Latif, G. et al. ^55^	BraTS dataset	SVM + LGG	Accuracy: 95.46%
Senan, E. M.. et al. ^56^		AlexNet + SVM	Accuracy: 95.10%
Vadhnani, S., & Singh, N. et al. ^57^	BraTS dataset	SVM(Linear)	Accuracy: 94%
Bodapati, N. et al. ^58^	BraTS dataset	K-means + ISVM	Accuracy: 95%
Huang, Z. et al. ^59^	BraTS dataset	CCN	Accuracy: 95.49%
Gumaei, A. et al. ^60^	BraTS dataset	GIST descriptor and ELM	Accuracy: 94.93%
Proposed Work	BraTS dataset	PCA-DenseNet121	Accuracy = 95.89% Precision = 94% Recall = 94% F1-Score = 94%

**Fig. 7:**
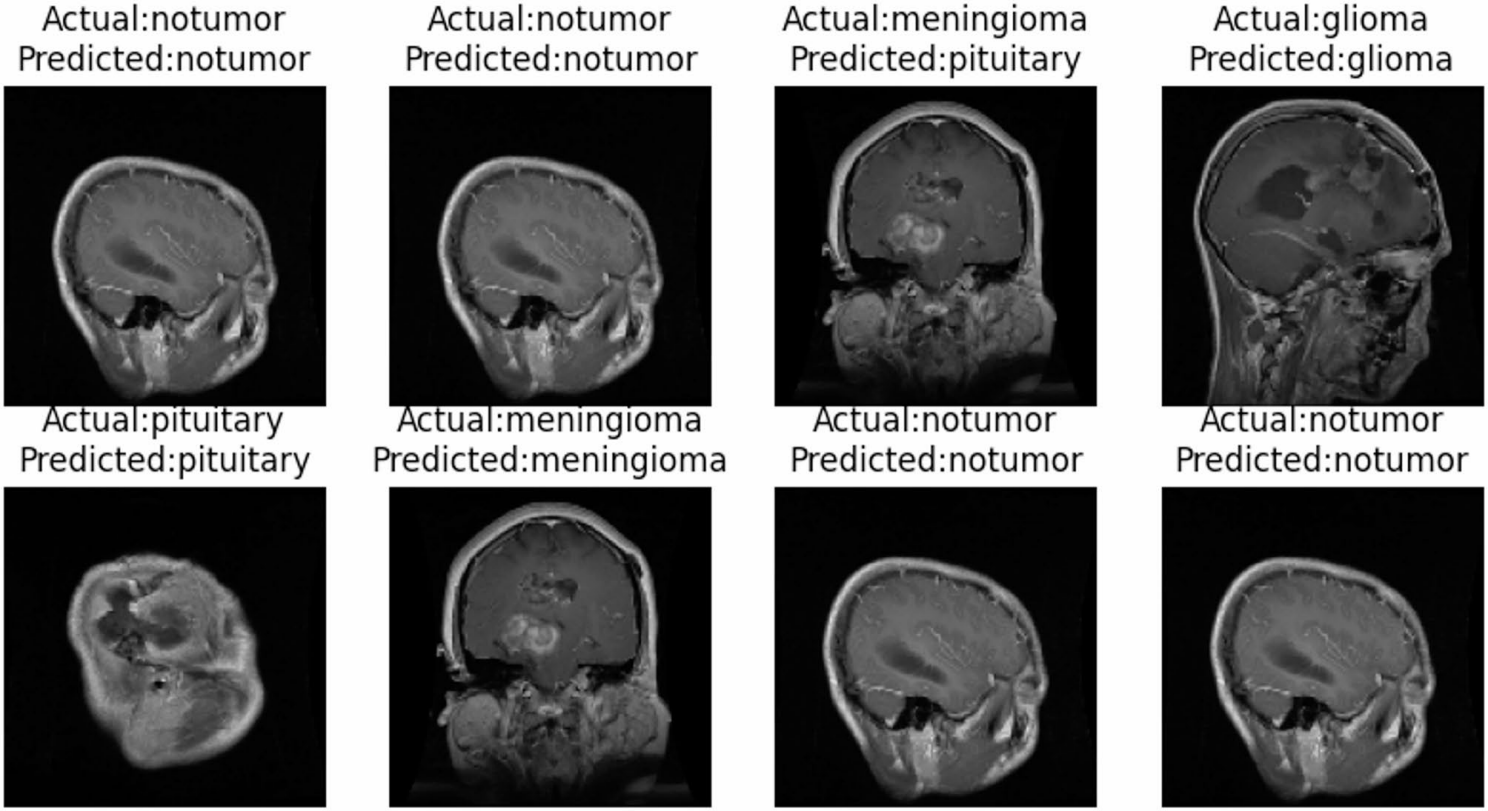
Comparative Study of Actual and Predicted Brain MRI images.

The PCA-DenseNet121 model outperformed previous approaches in classifying brain MRI images achieving a 95% accuracy and the precision, Recall and F1-score of 94%. It is not only showing better accuracy but it also maintains the lowest loss showing its ability to learn effectively from training image data and classify well to unseen image data. Figure 7 titled “Comparative Study of Actual and Predicted Brain MRI images,” visually presents the model’s predictions.

## Discussion

The experimental findings have demonstrated that the hybrid PCA-DenseNet121 framework offers an enhanced representation of brain MRI data. This architecture has routinely surpassed conventional standalone architectures across various performance criteria. The main advantage of this technology resides in its dual-domain feature extraction capability. The DenseNet-121 system proficiently acquires conceptual high-level spatial hierarchies through its dense interconnections. However, the deep networks frequently neglect the subtle textural differences. These nuances are clinically relevant for the diagnosis of disease at its earliest stages. The model addresses this restriction by incorporating GLCM, LTP, and CCV. It helps to explicitly integrate the statistical and topological characteristics to improve the accuracy of the classification, and the incorporation of the Color Coherence Vector (CCV) has been demonstrated to be crucial. The CCV is typically considered as a color-centric metric. This study modified it to accommodate grayscale intensity coherence. This adaptation allows the model to delineate tissue based on global spatial coherence. The method effectively differentiates between homogeneous regions of healthy white matter and heterogeneous intensity patterns. Such scattered patterns are frequently associated with malignant growths. GLCM properties further refine the classifier’s capabilities. Specifically, Contrast and Homogeneity identify sharp intensity transitions at tumor boundaries. These transitions have been often smoothed out during the pooling layers of deep convolutional neural networks. The Principal Component Analysis (PCA) serves as a bridge between the handcrafted features and the deep learning bottleneck. This step addresses the dimensionality curse inherent in high-dimensional datasets. The model reduces the handcrafted feature set to 249 principal components. This reduction maintains high variance. It also prevents the final fully connected layer from processing redundant data. Furthermore, this dimensionality reduction strategy resolves the structural incompatibility between 1D feature vectors and 2D image inputs. The application of transfer learning using model ImageNet weights offers an adequate basis through freezing the initial layers of the DenseNet-121.The model utilizes enhanced edge and shape detectors. Especially the final classification component necessitates modification for MRI-specific patterns. This method markedly lowers training waiting time, and also helps to reduce the likelihood of excessive overfitting. These factors have been especially crucial in situations with restricted medical datasets. This innovative synergy has been substantiated by the enhanced scores in accuracy, sensitivity, and the F1 score and a highly effective approach has been provided by integrating the automatic learning capabilities of CNNs with the deterministic accuracy of textural descriptors. This integration raises the bar for computer-aided diagnosis (CAD) in neuro-oncology.

## Conclusion

The PCA-DenseNet121 model standout as a highly effective and efficient solution for brain MRI classification combining deep learning and dimensionality reduction to provide better results. With preprocessing techniques such as image resizing, normalization, batch processing and label encoding, the model ensures optimized data handling and improve classification accuracy. The integration of DenseNet 121 model with PCA-based feature reduction accurately differentiates glioma, meningioma, pituitary and no tumor cases. It provides 95.89% accuracy, 94% precision, 94% recall and 94% F1-score. The model outperforms existing methods while maintaining the low loss, robust feature extraction and minimal misclassification. The training and validation curves show stable learning patterns showing that the model effectively overcomes overfitting through dropout layers and data shuffling techniques.

## Future scope

The proposed hybrid PCA-DenseNet121 framework demonstrates elevated accuracy in diagnosis. However, these constraints offer a strategic framework for the prospect of future research. The primary constraint involves, the reliance on one single data source. This reliance can inhibit the generalizability of the model across diverse clinical settings and various MRI hardware systems. Therefore, subsequent efforts will concentrate on cross-institution validation and the incorporation of real-world clinical data to ensure the resilience of the domain. The tri-fusion of GLCM, LTP, and CCV helps to capture intricate textural information. Although the manual feature extraction and PCA optimization impose burdens on the computational process. Optimizing these processes continues to be a goal for subsequent improvements. By integrating self attention techniques or vision transformers could facilitate the automatic assessment of the significant characteristics. These developments could help to enhance operational efficiency and the interpretability of the model. Moreover, the present utilization of K-means for segmentation has demonstrated vulnerability to border mistakes in low-contrast images. This constraint indicates a shift towards the deep learning architectures, such as U-Net, for precise delineation of regions of interest. Ultimately, broadening the framework parameters from binary detection to multi-class classification is required. The determination of molecular markers, particularly IDH mutation status, signifies a key achievement. This improvement will enable the conversion of the existing system into an all-encompassing clinical decision support instrument.

## Data Availability

The datasets used and/or analysed during the current study available from the corresponding author on reasonable request.

## Code availability

The relevant code with the manuscript is also available and would be available, if will be asked to do so later.
